# COVID 19 survivors : Feeling suicidal ?

**DOI:** 10.1192/j.eurpsy.2022.659

**Published:** 2022-09-01

**Authors:** K. Nourchene, E. Khelifa, B. Abassi, O. Maatouk, I. Bouguerra, S. Ben Aissa, L. Mnif

**Affiliations:** 1 Razi hospital, Skolly, Tunis, Tunisia; 2 Razi Hospital, F Adult Psychiatry Department, Manouba, Tunisia; 3 Errazi hospital-Mannouba, F, Mannouba, Tunisia; 4 Razi, Skolly, Manouba, Tunisia; 5 Hôpital Razi, Psychiatry F, Manouba, Tunisia

**Keywords:** survivor, Covid-19, Suicide

## Abstract

**Introduction:**

The COVID-19 pandemic is associated with several psychiatric manifestations leaving undoubtedly psychological consequences. However by escaping death ,do COVID-19 survivors present a higher risk for suicide ?

**Objectives:**

In this study, we aimed to explore suicidal risk among recovering COVID 19 patients .

**Methods:**

Our literature review was based on the PubMed interface and adapted for 2 databases: Science Direct and Google Scholar using the following combination ( suicide [MeSH terms]) AND (COVID-19 survivors[MeSH terms]).

**Results:**

Recovering COVID 19 patients are at risk for developping posttraumatic stress disorder , anxiety , depression and sleep abnormalities , especially in severe forms. Added to that ,cognitive impairment was largely described in COVID 19 causing judgment and reasoning decline. These manifestations would partially explain the suicidiality among survivors regardless to their medical hisotry. Nonetheless,many COVID-19 survivors experience persistent physical symptoms and psychiatric disorders leading to post-COVID syndrome which is associated with increased suicidal ideation and behavior In addition , social factors are considered as a suicide risk factor such as isolation ,loss of loved ones ,loss of job and economic instability .

**Conclusions:**

Over the course of illness , COVID 19 survivors may suffer from psychiatric and medical conditions leading to serieous suicide risk. Therefore ,suicide prevention interventions and appropriate medical management need to be provided to keep survivors alive .

**Disclosure:**

No significant relationships.

